# Physiological, Epigenetic, and Transcriptome Analyses Provide Insights into the Responses of Wheat Seedling Leaves to Different Water Depths under Flooding Conditions

**DOI:** 10.3390/ijms242316785

**Published:** 2023-11-26

**Authors:** Bo Li, Wei Hua, Shuo Zhang, Le Xu, Caixian Yang, Zhanwang Zhu, Ying Guo, Meixue Zhou, Chunhai Jiao, Yanhao Xu

**Affiliations:** 1 Hubei Key Laboratory of Food Crop Germplasm and Genetic Improvement and Key Laboratory of Crop Molecular Breeding, Food Crops Institute, Hubei Academy of Agricultural Sciences, Ministry of Agriculture and Rural Affairs, Wuhan 430064, China; boli@hbaas.com (B.L.);; 2 Institute of Crop and Nuclear Technology Utilization, Zhejiang Academy of Agricultural Sciences, Hangzhou 310021, China; huaweicau@hotmail.com; 3 Hubei Collaborative Innovation Centre for the Industrialization of Major Grain Crops, College of Agriculture, Yangtze University, Jingzhou 434025, China; 4 Tasmanian Institute of Agriculture, University of Tasmania, Newnham Drive, Launceston, TAS 7250, Australia

**Keywords:** *Triticum aestivum* L., DNA methylation, transcriptional analysis, water depth, flooding stress

## Abstract

Flooding stress, including waterlogging and submergence, is one of the major abiotic stresses that seriously affects the growth and development of plants. In the present study, physiological, epigenetic, and transcriptomic analyses were performed in wheat seedling leaves under waterlogging (WL), half submergence (HS), and full submergence (FS) treatments. The results demonstrate that FS increased the leaves’ hydrogen peroxide (H_2_O_2_) and malondialdehyde (MDA) contents and reduced their chlorophyll contents (SPAD), photosynthetic efficiency (*F_v_*/*F_m_*), and shoot dry weight more than HS and WL. In addition, FS increased catalase (CAT) and peroxidase (POD) activities more than HS and WL. However, there were no significant differences in the contents of H_2_O_2_, MDA, SPAD, and *F_v_*/*F_m_*, and the activities of superoxide dismutase (SOD) and POD between the HS and WL treatments. The changes in DNA methylation were related to stress types, increasing under the WL and HS treatments and decreasing under the FS treatment. Additionally, a total of 9996, 10,619, and 24,949 genes were differentially expressed under the WL, HS, and FS treatments, respectively, among which the ‘photosynthesis’, ‘phenylpropanoid biosynthesis’, and ‘plant hormone signal transduction’ pathways were extensively enriched under the three flooding treatments. The genes involved in these pathways showed flooding-type-specific expression. Moreover, flooding-type-specific responses were observed in the three conditions, including the enrichment of specific TFs and response pathways. These results will contribute to a better understanding of the molecular mechanisms underlying the responses of wheat seedling leaves to flooding stress and provide valuable genetic and epigenetic information for breeding flood-tolerant varieties of wheat.

## 1. Introduction

Flooding stress is a major ecological threat that restricts crop growth and yield in high-rainfall zones across the globe [1]. Based on the depth of the water table, flooding can be classified as waterlogging, when it is superficial and covers only the roots, or as submergence, when water completely covers the aerial plant tissues [2]. Flooding can cause the accumulation of reactive oxygen species (ROS), such as superoxide (O_2_^•−^) and hydrogen peroxide (H_2_O_2_), increasing lipid peroxidation and resulting in plant cell damage [3,4]. Flooding also results in a reduction in stomatal conductance, the CO_2_ assimilation rate, photosynthesis rate, and nutritional imbalance [5]. With the onset of climate change, the average and extreme precipitation intensities and the frequency of extremely heavy precipitation have significantly increased [6]. Therefore, understanding the response mechanisms of plants to flooding stress has important implications for developing flood-tolerant varieties and promoting their effective adaptation to climate change.

In recent decades, the physiological and molecular mechanisms of adaptation to flooding stress have been reported [7,8,9,10,11,12]. For example, increases in the contents of superoxide dismutase (SOD), peroxidase (POD), catalase (CAT), and other antioxidant enzymes, have been suggested as a key strategy through which plants can effectively resist waterlogging stress [8]. For example, γ-aminobutyric acid was found to enhance waterlogging tolerance in maize by increasing the activities of several antioxidant enzymes (SOD, POD, CAT, etc.) [13]. A transcriptomic analysis showed that Chinese wingnut plants enhanced their waterlogging tolerance by increasing their synthesis of alpha-linolenic acids and flavonoids and activating plant hormone signaling pathways [8]. In cucumber, waterlogging-induced differentially expressed genes were especially related to enhanced glycolysis, adventitious root development, and amino acid metabolism [11]. In *Phalaris arundinacea*, waterlogging-stress-induced differentially expressed genes were mainly involved in carbohydrate metabolism, hormone signaling regulation, and the scavenging of reactive oxygen species [12]. Moreover, TFs, including MYB, bHLH, NAC, WRKY, ERF, and bZIP, are also reported to be the major regulators involved in waterlogging tolerance [12]. For instance, the ERFs of group VII are well known for facilitating ethylene signal transduction and enhancing waterlogging tolerance in maize [14] and wheat [15].

Epigenetic alterations have been found to be associated with abiotic stresses in crop species [16,17,18,19]. DNA methylation is one of the critical epigenetic mechanisms for the regulation of gene expression under abiotic stresses [17,18,20,21]. In wheat, DNA demethylation significantly increases waterlogging-related gene expression in tolerant genotypes under hypoxic stress, such as the expression of *ERF1*, *ACC1*, and *CKX2.3* [18]. In rice, DNA methylation in response to drought stress regulates the expression of unique genes responsible for drought stress tolerance [16,19]. Furthermore, the response of DNA methylation to stress is also related to the type of stress. For example, total DNA methylation increases under drought stress in sesame but decreases under waterlogging stress [17]. These studies show that epigenetic regulation is important in the abiotic stress responses in plants. However, the potential mechanisms underlying epigenetic regulation under flooding stress remain largely unclear.

In recent years, water depth has been recognized as one of the important factors limiting the growth and development of flooded plants [22,23,24]. For example, the leaves of mulberry seedlings under shallow submergence were healthy, while the leaves of mulberry seedlings treated with half submergence and full submergence showed waterlogging symptoms, to varying degrees, in their middle [22]. Partially submerged *Paspalum dilatatum* plants showed a reduction in the starch concentration in their leaves, but their biomass was unaffected, whereas completely submerged plants did not survive [23]. All *Melilotus siculus* accessions were able to reorient petioles towards the vertical axis under both partial and full submergence [24]. However, petiole extension rates were maintained under the partial submergence treatment and decreased under the full submergence treatment. Moreover, the sugar contents of *Melilotus siculus* leaflets rose during partial submergence but were depleted during full submergence [24]. These studies implied that different performance and response mechanisms exist for the response to flooding stresses at different water depths, but the underlying adaptation mechanisms remain largely unknown.

Wheat (*Triticum aestivum* L.) is an important food crop, and major source of calories, that is grown worldwide [25]. In the case of wheat, the seedling stage is one of the important stages at which waterlogging is most detrimental to wheat yields, after germination [9]. Approximately 15–20% of the total wheat cropping area is under threat of flooding, which could reduce the global grain yield by up to 43% [26,27]. Thus far, physiological, transcriptional, and epigenetic regulatory responses to waterlogging/hypoxic stress in wheat have been investigated [9,15,18]. However, whether different flooding depths affect the physiology, transcription, and epigenetics of wheat is a question that is yet to be fully answered, and one that is related to the screening and evaluation of plant flooding tolerance and the breeding of resistant varieties. Thus, in this study, to gain insights into the molecular responses of wheat to flooding, three types of flooding stress treatments, based on water depth, were designed: soil surface submergence (the water was superficial and covered only the roots and soil surface), half submergence (the water covered half of the plant), and full submergence (the water completely covered the plant tissues). We systematically investigated the changes in the physiology, epigenetic regulation (DNA methylation), and genomic transcription of wheat seedling leaves under controlled conditions and three types of flooding conditions. This study aims to provide a comprehensive research framework for understanding the physiological changes, epigenetic regulation, and global transcription in wheat under flooding stress, which is of great significance for the genetic improvement and resistance evaluation of flood-resistant wheat varieties.

## 2. Results

### 2.1. The Distinct Effects of FS and HS/WL on Biomass and Photosynthesis

**Figure 1:**
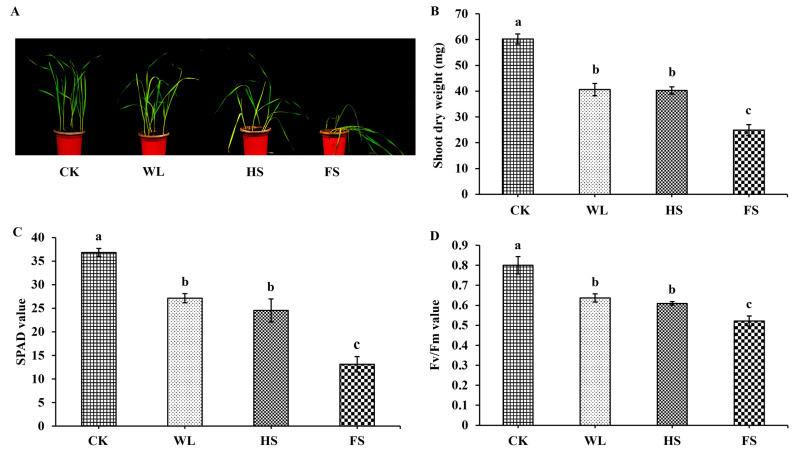
Biomass and photosynthesis analysis of Efumai 1 seedlings in the control and three flooding treatments. (**A**) Images of Efumai 1 plants after a 7-day treatment under CK, WL, HS, and FS conditions; (**B**) shoot dry weight; (**C**) SPAD value; and (**D**) *Fv*/*Fm* value. Significant differences were assessed using ANOVA with *p* < 0.05; The different letters above the bars indicated significant differences (*p* < 0.05). Three independent biological replicates for each treatment were performed and six pots were established for each replicate.

After a 7-day treatment, the Efumai 1 plants under FS treatment showed wilting and yellowing, whereas the plants under the WL and HS treatments had slightly wilted leaves (Figure 1A). The shoot dry weight significantly decreased by 58.26% under the FS treatment compared to the control plants, followed by the HS (40.10%) and WL (38.70%) treatments (Figure 1B). Their SPAD values significantly decreased by 26.33%, 33.41%, and 64.41%, and their *Fv*/*Fm* values significantly decreased by 20.46%, 23.85%, and 34.87%, under the WL, HS, and FS treatments, respectively (Figure 1C,D). Interestingly, no differences were observed between WL and HS.

### 2.2. Physiochemical Changes under the Three Flooding Conditions

**Figure 2:**
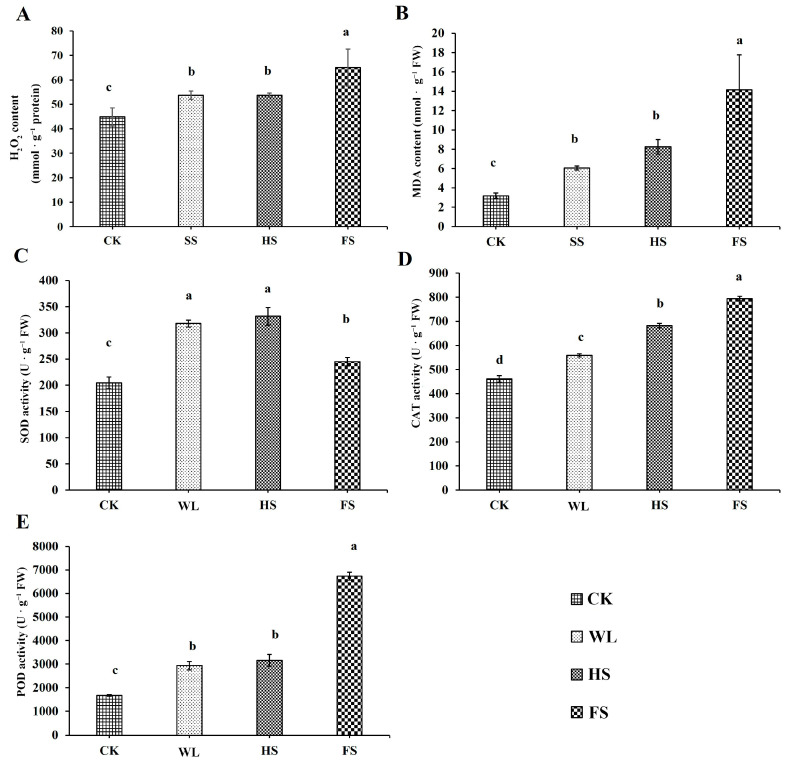
Physiological responses of wheat seedlings under the control and three flooding treatments. (**A**) H_2_O_2_ content; (**B**) MDA content; (**C**) SOD activity; (**D**) CAT activity; and (**E**) POD activity. Values are expressed as mean ± SD. The different letters above the bars indicated significant differences using ANOVA with *p* < 0.05.

The H_2_O_2_ content of each sample was measured, and the results show that the FS treatment resulted in a significant increase in H_2_O_2_ content (by 44.85%), followed by the WL (19.72%) and HS (19.60%) treatments, compared with the control (CK) (Figure 2A). Similarly, the MDA content significantly increased under the three flooding conditions, and the highest MDA content was found in the FS treatment (344.62% increase), followed by the HS (159.07%) and WL (90.10%) treatments (Figure 2B). Compared with CK, the highest CAT (72.18% increase) and POD (303.13%) activities were also found in the FS treatment (Figure 2D,E), while the highest SOD activity was identified in the WL (55.44%) and HS (62.21%) treatments (Figure 2C). Notably, except for CAT, the H_2_O_2_ and MDA contents and SOD activities did not differ between the HS and WL treatments (Figure 2).

### 2.3. Global Changes in DNA Methylation under the CK, WL, HS, and FS Conditions

A total of 453 clear and reproducible bands were successfully obtained under the CK, WL, HS, and FS conditions (Figure S1). Most of the CCGG sites were shown to be largely methylated, with values ranging between 86.75% and 93.38% (Table 1), and there was a greater number of fully methylated bands (average 65.45%) (types Ⅲ and Ⅳ) than hemi-methylated bands (type Ⅱ) (average 23.95%). Compared with the CK (88.30%), the total DNA methylation level was slightly increased to 89.18% and 93.38% under the WL and HS conditions, respectively. This ratio decreased to 86.75% under the FS treatment (Table 1).

To further investigate the difference in wheat DNA methylation in response to the WL, HS, and FS treatments, a total of 16 alternative band patterns between the CK and the flooding stress treatments were identified, which could be classified into three groups: no change, hypomethylation, and hypermethylation (Table 2). Compared with the CK, 69.50% of the detected loci showed an altered DNA methylation status under the HS conditions, followed by 51.11% under FS, and 43.79% under WL. The percentages of bands hypermethylated were 27.09% and 52.52% under the WL and HS treatments, respectively, higher than that under FS (25.33%). More hypomethylation events were detected in the FS (25.78%) than in the WL (16.70%) and HS conditions (16.97%). These results suggest the divergent reprogramming of the methylation pattern under the different types of flooding stresses.

### 2.4. Analysis of DEGs in Wheat in Response to the Three Types of Flooding Stress

After filtering, an average of 81.90 million clean reads were obtained from each sample. The average GC value was 57.00%. The Q30 percentage of the 12 libraries varied from 93.38% to 93.75%. More than 84% of the clean reads were uniquely mapped to the wheat reference genome sequence (Table S1). A principal component analysis (PCA) of all the genes in the 12 libraries revealed a clear separation between the control and flooding treatments. However, the WL- and HS-treated samples were closest to each other, indicating the highest degree of similarity in their transcription patterns (Figure S2A). Furthermore, a Pearson correlation analysis showed a high correlation among the biological replicates (the Pearson correlation coefficient ranged from 0.97 to 0.99) (Figure S2B). These results indicate the good reproducibility and quality of the RNA-seq data.

**Figure 3:**
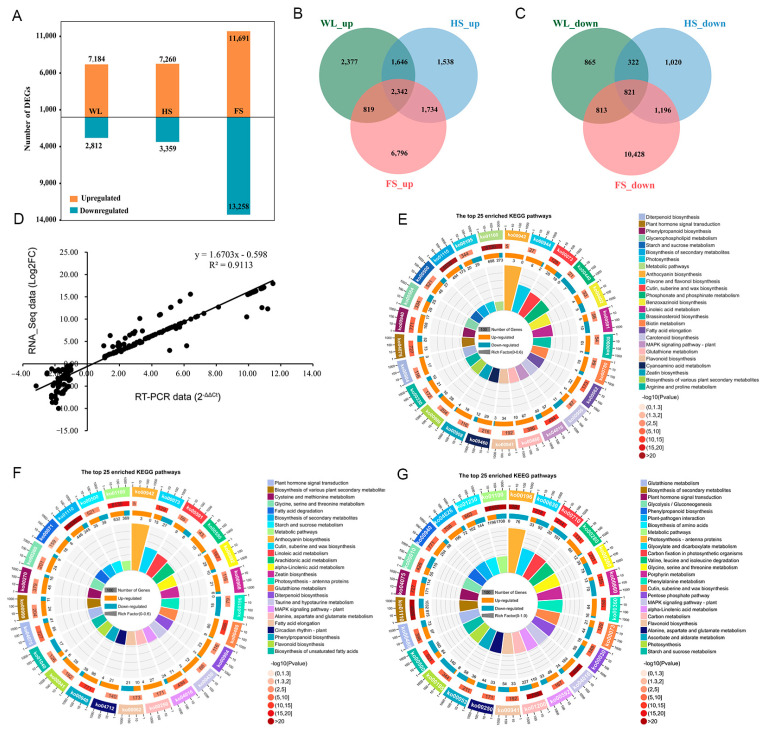
Overview of the effects of flooding treatments on gene expression. (**A**) Number of DEGs in the three comparisons. (**B**) Overlapping upregulated DEGs in the three comparisons. (**C**) Overlapping downregulated DEGs in the three comparisons. (**D**) Correlation analysis of differentially expressed genes between RT-PCR analysis (2^−ΔΔCt^ value) and RNA-seq experiment (Log_2_ fold change value). The top 25 significantly enriched KEGG pathways of DEGs induced by WL (**E**), HS (**F**), and FS (**G**) treatments, from the outside to the inside. The first circle represents the top 25 enrichment pathways, and the number outside the circle is the coordinate ruler of the number of genes. The second circle represents the number and −log10 (*p* value) of background genes in this pathway. The third circle represents the number of upregulated and downregulated DEGs in this pathway. The fourth circle represents the value of the Rich Factor in each pathway.

Differential expression analysis between the flooding-treated and control samples revealed a total of 9996, 10,619, and 24,949 DEGs in the WL, HS, and FS conditions, respectively (Figure 3A and Table S2). A Venn diagram showed that a total of 32,708 DEGs (17,252 upregulated and 15,456 downregulated) were identified across the three flooding conditions (Figure 3B,C). A cross-comparison between the different gene sets showed that the highest proportion of DEGs (52.66%) was unique to the FS treatment, while the WL (9.88%) and HS (7.82%) treatments exhibited fewer uniquely expressed genes. Furthermore, 2342 upregulated and 821 downregulated DEGs were detected under all three flooding conditions, which suggested that these genes might play an important role in the functioning of wheat under flooding stress. To validate the reliability of the expression profiles obtained using RNA-Seq, sixty DEGs were randomly selected to perform qRT–PCR on. Pearson’s correlation coefficients showed that the qRT–PCR and RNA sequencing data for these genes were highly correlated (*r* = 0.91) (Figure 3D).

### 2.5. KEGG Enrichment Analysis of the WL-, HS- and FS-Induced DEGs

**Figure 4:**
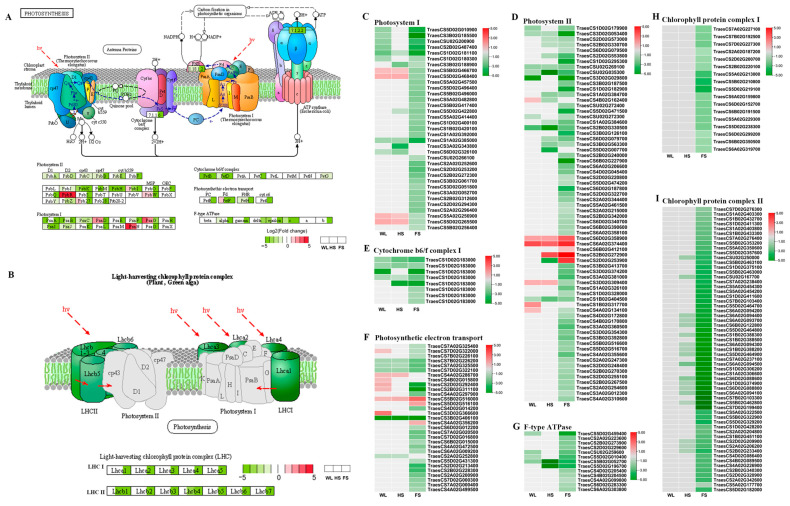
DEGs related to the photosynthesis pathways. The KEGG pathway map of ‘photosynthesis’ (**A**) and ‘photosynthesis-antenna protein’ (**B**). The DEG expression pattern was used to annotate and generate a corresponding map. The green box with gene symbols denotes downregulated expression in the chlorosis group, while the red box denotes upregulated expression. The genes that were not significantly altered are displayed with a white box. (**C**–**G**) Expression profiles (Log2 fold change) of genes related to ‘photosynthesis’ pathway. (**H**,**I**) Expression profiles (Log2 fold change) of genes related to ‘photosynthesis-antenna proteins’ pathway.

KEGG enrichment analysis showed that a total of 36, 37, and 66 pathways were significantly enriched (*p* value < 0.05) under the WL, HS, and FS conditions, respectively (Tables S3–S5). The top 25 significantly enriched KEGG pathways are presented in Figure 4E–G, in which 24 of the enriched pathways—including the ‘biosynthesis of secondary metabolites’, ‘metabolic pathways’, ‘phenylpropanoid biosynthesis’, ‘plant hormone signal transduction’, ‘starch and sucrose metabolism’, ‘flavonoid biosynthesis’, and ‘photosynthesis’—were common to all three flooding treatments. Moreover, a number of DEGs were enriched in four pathways unique to the WL treatment—‘brassinosteroid biosynthesis’, ‘biotin metabolism’, ‘ether lipid metabolism’, and ‘glycosaminoglycan degradation’ (Figure 4E and Table S3)—whereas three pathways were specific to the HS treatment—‘taurine and hypotaurine metabolism’, ‘circadian rhythm-plant’, and ‘selenocompound metabolism’ (Figure 4F and Table S4). Moreover, 32 specific pathways were significantly enriched in response to the FS treatment, including ‘carbon metabolism’, ‘glyoxylate and dicarboxylate metabolism’, ‘carbon fixation in photosynthetic organisms’, ‘porphyrin and chlorophyll metabolism’, and ‘peroxisome’ (Figure 4G and Table S5).

Among the WL-induced DEGs, a total of 7184 upregulated genes were identified and significantly enriched in 36 KEGG pathways, including the ‘biosynthesis of secondary metabolites’, ‘metabolic pathways’, ‘phenylpropanoid biosynthesis’, ‘glutathione metabolism’, and ‘fatty acid elongation’ (Table S6). Eighteen KEGG pathways were significantly enriched in the 2812 downregulated genes, including ‘Plant hormone signal transduction’, ‘MAPK signaling pathway-plant’, ‘Cutin, suberine and wax biosynthesis’, and ‘Photosynthesis’ (Table S7). Under the HS condition, 7260 upregulated and 3359 downregulated genes were identified (Figure 3A). KEGG pathway analysis showed that these upregulated DEGs were significantly enriched in 33 pathways, including the ‘biosynthesis of secondary metabolites’, ’glutathione metabolism’, ‘phenylpropanoid biosynthesis’, and ‘plant hormone signal transduction’ (Table S8). A total of 19 KEGG pathways were significantly enriched in the 3359 downregulated genes, including the ‘Biosynthesis of secondary metabolites’, ‘Metabolic pathways’, ‘Photosynthesis-antenna proteins’, and ‘Photosynthesis’ (Table S9). Under the FS condition, a total of 11,691 up- and 13,258 downregulated genes were identified and significantly enriched in 50 and 39 KEGG pathways, respectively. Among these pathways, the genes involved in the ‘biosynthesis of secondary metabolites’, ‘phenylpropanoid biosynthesis’, and ‘plant hormone signal transduction’ were mainly upregulated (Table S10), and the genes involved in ‘photosynthesis-antenna proteins’, ‘photosynthesis’, and the ‘carbon fixation in photosynthetic organisms’ were mainly downregulated (Table S11).

### 2.6. GO Enrichment Analysis of the WL-, HS- and FS-Induced DEGs

GO classification analysis showed that there were similar processes enriched in the wheat leaves exposed to SS, HS, and FS, including metabolic process and cellular process in the biological process category, binding and catalytic activity in the molecular function category, and membrane and membrane parts in the cellular component category. However, the genes enriched in these GO terms were mainly upregulated under SS and HS, while they were mainly downregulated under FS (Figure S3). Furthermore, GO enrichment analysis showed that 116 and 64 GO terms were significantly enriched in the upregulated and downregulated genes under WL conditions, respectively (Tables S12 and S13). Of these, ‘catalytic activity’, ‘extracellular region’, ‘cell wall’, ‘external encapsulating structure’, and ‘hydrolase activity, acting on glycosyl bonds’ were the top five significantly enriched GO terms for the genes upregulated under WL conditions (Table S12), and ‘glucosidase activity’, ‘sucrose alpha-glucosidase activity’, ‘pyruvate kinase activity’, ‘potassium ion binding’, and ‘alkali metal ion binding’ were the top five significantly enriched GO terms for the genes downregulated under WL conditions (Table S13). A total 83 and 143 GO terms were significantly enriched in the HS-induced upregulated and downregulated genes, respectively (Tables S14 and S15). Of these, ‘transferase activity, transferring hexosyl groups’, ‘solute:cation symporter activity’, ‘solute: proton symporter activity’, ‘symporter activity’, and ‘transferase activity, transferring glycosyl groups’ were the top five significantly enriched GO terms in the upregulated genes (Table S14). Meanwhile the ‘lipid biosynthetic process’, ‘catalytic activity’, ‘transferase activity, transferring acyl groups other than amino-acyl groups’, ‘transferase activity, transferring acyl groups’, and ‘fatty acid biosynthetic process’ were the top five significantly enriched GO terms for the downregulated genes (Table S15). Under the FS condition, a total of 153 and 194 GO terms were significantly enriched in the upregulated and downregulated genes, respectively (Tables S16 and S17). Of these, ‘protein kinase activity’, ‘catalytic activity’, ‘kinase activity’, ‘phosphotransferase activity, alcohol group as acceptor’, and ‘transferase activity’ were the top five significantly enriched GO terms for the upregulated genes (Table S16), and ‘photosynthesis’, ‘thylakoid plastid’, ‘plastid part’, and ‘thylakoid membrane’ were the top five significantly enriched GO terms for the upregulated genes (Table S17).

### 2.7. Analysis of the DEGs Related to the Photosynthesis Pathway in Response to WL, HS, and FS Stresses

Two photosynthesis pathways, ‘photosynthesis-antenna proteins’ and ‘photosynthesis’, were significantly enriched in all flooding conditions in the KEGG analysis (Tables S3–S5), while ‘photosynthesis-antenna proteins’ were only significantly enriched under HS and FS (Tables S3–S5). In total, 155 DEGs were related to KEGG photosynthesis pathways (Figure 4A and Table S6), including 35 genes related to photosystem I (PSI) (Figure 4C), 68 genes related to photosystem II (PSII) (Figure 4D), 8 genes related to cytochrome b6/f complex (Figure 4E), 31 genes related to photosynthetic electron transport (Figure 4F), and 13 genes related to F-type ATPase (Figure 4G). A total of 76 DEGs were involved in the ‘photosynthesis-antenna proteins’ pathway (Figure 4B and Table S7), including 17 genes related to light-harvesting chlorophyll protein complex I (LHCI) (Figure 4H) and 59 genes related to LHCII (Figure 4I). Most of the DEGs involved in the both the ‘photosynthesis-antenna proteins’ and ‘photosynthesis’ pathways were significantly downregulated, especially under the FS treatment (Figure 4).

### 2.8. Analysis of the DEGs Related to Phenylpropanoid Biosynthesis and Antioxidant Pathways in Response to WL, HS, and FS Stresses

**Figure 5:**
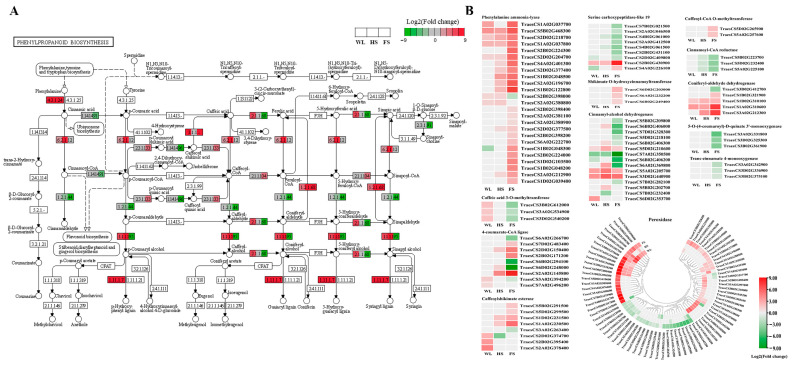
DEGs involved in the phenylpropanoid biosynthesis pathway. (**A**) The location of DEGs in phenylpropanoid biosynthesis pathway. (**B**) The expression pattern of the phenylpropanoid-biosynthesis-related genes.

Since the phenylpropanoid biosynthesis pathway was significantly enriched under the three types of flooding stresses, the expression patterns of the genes involved in this pathway were further compared across the three flooding stress treatments (Figure 5A). A total of 162 DEGs related to phenylpropanoid biosynthesis were detected under the three flooding conditions (Figure 5B and Table S18): 74 peroxidases, 25 phenylalanine ammonia-lyases, 15 cinnamyl-alcohol dehydrogenases, 9 4-coumarate-CoA ligases, 9 serine carboxypeptidase-like 19 genes, 8 caffeoyl shikimate esterases, and 5 coniferyl-aldehyde dehydrogenases (Figure 5B and Table S18). Of the phenylpropanoid-related genes, 21 genes were co-upregulated by WL, HS, and FS: 11 peroxidases, 4 phenylalanine ammonia-lyases, 3 cinnamyl-alcohol dehydrogenases, 2 coniferyl-aldehyde dehydrogenases, and 1 serine carboxypeptidase-like 19 (Figure 5B). Interestingly, TraesCS2B02G398000, encoding a phenylalanine ammonia-lyase, was upregulated under the WL and HS treatments, while it was downregulated under the FS treatment (Figure 5B). Furthermore, 16 DEGs, including 12 peroxidases, 2 caffeoyl shikimate esterases, 1 4-coumarate-CoA ligase, and 1 cinnamyl-alcohol dehydrogenase, were uniquely upregulated under the WL treatment, while 4 genes, including 2 peroxidases and 2 cinnamyl-alcohol dehydrogenases, were exclusively expressed under the HS treatment. In addition, 66 DEGs—28 peroxidases, 10 phenylalanine ammonia-lyases, 4 serine carboxypeptidase-like 19 proteins, 4 cinnamyl-alcohol dehydrogenases, 3 caffeoylshikimate esterases, 3 shikimate O-hydroxycinnamoyltransferases, and 3 5-O-(4-coumaroyl)-D-quinate 3′-monooxygenases—were only differentially expressed under the FS treatment (Figure 5B and Table S18).

The genes encoding the enzymes involved in reactive oxygen species (ROS) metabolism, mainly glutathione S-transferase (GST, 181), glutathione peroxidase (GPX, 4), ascorbate peroxidase (APX, 7), SOD (8), and CAT (7), were also differentially expressed (Table S19). A total of 181 GSTs were differentially expressed, and over half of these were upregulated, accounting for 95.46% (63 of 69), 92.39% (85 of 92), and 62.84% (93 of 148) of the GSTs induced by the WL, HS, and FS treatments, respectively. Thirty-one GSTs were co-upregulated under all treatments. Moreover, 10 (8 upregulated and 2 downregulated), 15 (12 upregulated and 3 downregulated), and 68 (33 upregulated and 35 downregulated) specific GSTs were regulated by WL, HS, and FS, respectively. Seven genes encoding CATs were identified and upregulated under all the conditions, but the majority of them were induced by the FS treatment. On the other hand, a total of seven APXs, four GPXs and eight SODs were identified and mainly downregulated under the three flooding treatments. Two SODs and two GPXs were co-upregulated and co-downregulated under the three stress conditions, respectively. Five SODs, five APXs, and two GPXs were exclusively regulated by FS (Table S19).

### 2.9. Expression of the Plant Hormone Signal Transduction Pathway and Transcription Factor Genes in Response to Different Flooding Stresses

**Figure 6:**
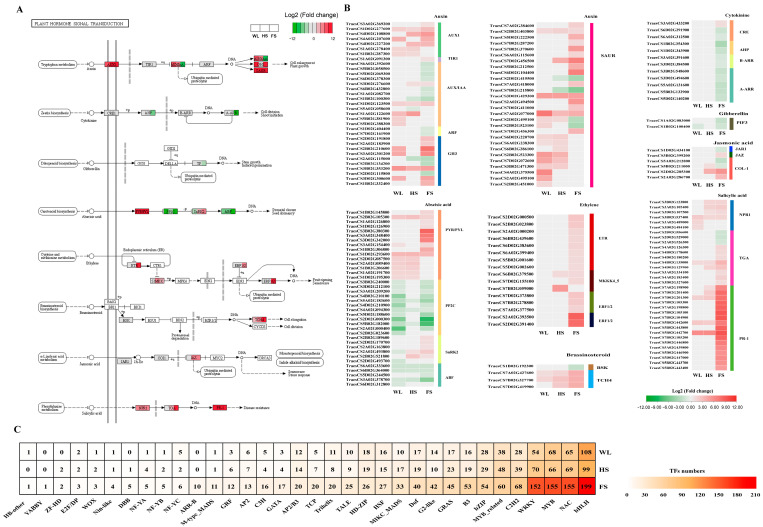
DEGs (**A**,**B**) and transcription factors (**C**) involved in the plant hormone signal transduction pathway.

In addition to the phenylpropanoid biosynthesis pathway, KEGG enrichment results also showed that genes related to the plant hormone signal transduction pathway were significantly enriched in all the flooding treatments. A total of 173 DEGs were identified to be involved in eight plant hormone signal transduction pathways, including those for auxin (IAA), cytokinin (CTK), gibberellin (GA), abscisic acid (ABA), ethylene (ETH), brassinosteroid (BR), jasmonic acid (JA), and salicylic acid (SA) (Figure 6A and Table S20).

For the IAA signal, a total of 61 DEGs were identified, including 28 SAUR families (SAUR), 13 auxin-responsive IAA (AUX/IAA), 13 auxin-responsive GH3 gene families (GH3), 7 auxin influx carriers (AUX1 LAX family) (AUX1), 2 auxin response factors (ARF), and 1 transport inhibitor response 1 (TIR1) protein (Figure 6B and Table S4). Among these IAA-related genes, seven genes, including four SAURs, one AUX1, one AUX/IAA, and one GH3, were coregulated via the WL, HS and FS treatments. Moreover, two AUX/IAAs and three SAURs were exclusively regulated by the WL treatment, and one AUX/IAA and one SAUR were uniquely regulated by HS (Figure 6B and Table S20). However, 26 DEGs, including 11 SAURs, 6 AUX/IAAs, 5 GH3s, 2 ARFs, 1 AUX1, and 1 TIR1, were only differentially expressed under the FS condition (Figure 6B and Table S20).

A total of 12, 2, 16, and 6 DEGs were identified in the CK, GA, ETH, and JA signaling pathways, respectively, and most of these were only differentially expressed under FS treatment (Figure 6B and Table S20). Most of the genes in the CTK, GA, and JA signaling pathways were downregulated, while the 16 ETH-related DEGs were upregulated under the FS treatment.

For ABA signaling, 38 genes, including 15 abscisic acid receptor PYR/PYL family genes (PYR/PYLs), 12 protein phosphatase 2Cs (PP2Cs), 6 serine/threonine-protein kinase SRK2s (SnRK2s), and 5 ABA responsive element binding factors (ABFs), were identified. Of these, two PYR/PYLs, three PP2Cs, and three ABFs were coregulated under the three flooding conditions (Figure 6B and Table S20). On the other hand, three genes, encoding a PP2C, an SnRK2, and an ABF, were only downregulated under WL conditions, while nine DEGs, including seven PYR/PYLs and two SnRK2s, were exclusively upregulated under the FS condition (Figure 6B and Table S20).

For the BR signal, four genes, encoding one BR-signaling kinase (BSK) and three xyloglucan–xyloglucosyl transferase TCH4 proteins (TCH4s), were detected (Figure 6B and Table S20). Two TCH4s (TraesCS7A02G427600 and TraesCS7B02G327700) were co-upregulated under all the flooding conditions. TraesCS1B02G192300, encoding a BSK, was only downregulated under the FS treatment (Figure 6B and Table S20).

For the SA signal, 34 DEGs, including 6 regulatory NPR1 proteins (NPR1s), 11 TGA transcription factors, and 17 pathogenesis-related protein 1s (PR-1s), were detected in this study (Figure 6B and Table S20). Most of these genes were upregulated under one or more flooding conditions. Five genes, including one NPR1 and four PR-1s, were co-upregulated in all flooding conditions, while two NPR1s were only differentially expressed under the WL condition. Nine TGAs and twelve PR-1s were only downregulated or upregulated under the FS treatment. Furthermore, three NPR1s, one TGA, and two PR-1s were co-upregulated under both the HS and FS conditions (Figure 6B and Table S20).

A total of 1754 differentially expressed TFs were identified under the WL (618), HS (696), and FS (1447) treatments, and these TFs were classified into 31, 32, and 35 families, respectively (Figure 6C and Table S21). Overall, the bHLH TF family was the most abundant, followed by the NAC, MYB, WRKY, and AP2/ERF families, under the three flooding conditions (Figure 6C). To gain insights into the regulation of TFs for different types of flooding stress, a cross-comparison was performed and showed that 271 TFs were commonly regulated under the three flooding treatments, including the bHLH (48), AP2/ERF (29), WRKY (26), NAC (25), MYB_related (23), and MYB (23) TFs (Figure 6C and Table S21). Under the WL treatment, 94 unique TFs belonging to 23 TF families were identified, including the bHLH (17), NAC (15), MYB (12), and WRKY (7) TFs. Furthermore, 113 TFs, involving 23 families, were uniquely induced via the HS treatment, including 15 bHLHs, 12 C2H2s, 10 WRKYs, and 8 AP2/ERFs. Additionally, 811 TFs were specifically induced via the FS treatment and classified into 33 families, including 101 bHLHs, 87 NACs, 844 MYBs, 79 WRKYs, and 65 AP2/ERFs. Moreover, two TF families, ARR-B and YAABY, were only identified under the FS treatment (Figure 6C and Table S21).

## 3. Discussion

### 3.1. The Submergence of Functional Leaves Was the Key Factor Determining Photosynthetic Efficiency after Submergence

Flooding usually reduces the rate of photosynthesis in plants [7]. Chlorophyll fluorescence is suggested to be a sensitive indicator of the stress-induced damage to photosystem Ⅱ, and a reduction in *F_v_/F_m_* is a good indicator of the photosynthetic impairment resulting from waterlogging stress [4]. Previous studies have found that waterlogging stress significantly reduces SPAD and *F_v_/F_m_* values [4,10]. Moreover, waterlogging stress damages the PSII reaction center of mulberry seedlings (*F_o_*, *F_m_*, and *F_v_*/*F_o_*), reducing their ability to accept electrons, and the degree of damage is proportional to the submergence depth [22]. Similar results were also observed in the present study, in which FS induced a greater reduction in the SPAD and *F_v_/F_m_* values of wheat leaves and led to a higher loss of biomass than HS and WL (Figure 1), which is consistent with the results of the transcriptome analysis, where the DEGs involved in ‘photosynthesis-antenna proteins’ and ‘photosynthesis’ pathways were significantly downregulated, especially under the FS treatment (Figure 4). Furthermore, more DEGs involved in the photosystem II process were downregulated than in other sub-pathways of the ’photosystem’ pathway (Figure 4D). Meanwhile, no significant difference in these parameters was detected between the WL and HS treatments (Figure 1C,D), which is consistent with a previous study showing that the biomass (shoot and root) of *Paspalum dilatatum* plants did not change after 30 days of waterlogging and partial submergence treatments, whereas completely submerged plants did not survive [23]. Additionally, a significant difference in plant weight between partial and full submergence conditions was also detected in *Melilotus siculus* plants, and the plant weight showed a greater reduction under the FS condition than under the partial submergence condition [24]. These results may indicate that the effect of flooding on phenotypic changes may be the same when functional leaves are not submerged.

### 3.2. The Role of Antioxidant Mechanisms in Response to the Three Types of Flooding Conditions

Flooding conditions lead to hypoxic and anoxic conditions for plants and induce the production of ROS in plant cells, and ROS can directly attack membrane lipids, leading to the accumulation of MDA [4,7,13]. MDA is commonly used as a marker of lipid peroxidation, reflecting the degree of cell membrane damage incurred in response to different environmental stresses [28]. For instance, waterlogging stress significantly increased the contents of O_2_^•−^, ^•^OH, and H_2_O_2_ in maize leaves, leading to an accumulation of MDA [13]. Wang et al. (2020) found that, compared to salt stress, saline–alkali stress more significantly upregulated MDA levels in *Triarrhena sacchariflora* leaves, indicating that saline–alkali stress caused greater harm to yellow horn seedlings [29]. In the present study, a significant increase in H_2_O_2_ content was observed under the FS treatment, leading to a higher content of MDA in wheat leaves, which clearly indicated the existence of oxidative stress and greater damage in response to FS stress than when plants were exposed to WL and HS stresses (Figure 1 and Figure 2).

Antioxidant enzymes, including SOD, POD, CAT, APX, and GPX, play vital roles in eliminating ROS [4,7,30]. The high activities of these enzymes under stress can improve stress tolerance in plants [4,13,30,31]. SOD catalyzes the conversion of peroxide anions to H_2_O_2_ and O_2_, whereas POD, CAT, and APX catalyze the conversion of H_2_O_2_ to oxygen and water [29]. In this study, SOD, POD, and CAT activities significantly increased under all the flooding stresses compared to the control. The result was similar to those of previous reports on soybean [32], peach [4], maize [13], *Triarrhena sacchariflora* [7], and wheat [30] under waterlogging stress. Thus, the expression of these antioxidant enzymes may be induced by flooding stress, and these enzymes may be critical for the survival of wheat leaves under flooding conditions [7]. Interestingly, a discrepancy between the gene expression levels and the corresponding enzyme activities was observed. For example, the activity of SOD increases only slightly, but the corresponding gene expression dramatically increases (Figure 2C and Table S19). These results may be due to the fact that the translation of expressed mRNA into active proteins is a complicated process that may be affected by multiple factors, such as post-transcriptional modifications, the stoichiometric balance of protein biosynthesis, and protein degradation [33]. The expressed mRNA is not necessarily translated into a similar amount of detected enzyme activity [34]. The activities of CAT and POD under FS were significantly higher than those under HS and WL. Furthermore, in terms of the H_2_O_2_ and MDA contents and SOD and POD activities, there was no significant difference between the WL and HS treatments. Similar results were also observed for the expression of ROS-scavenging-related genes (Tables S18 and S19). These results suggest that the ROS regulation mechanisms induced by WL/HS and FS are different.

### 3.3. Genes Related to Phenylpropanoid Biosynthesis Were Differentially Regulated in Response to Different Flood Stresses in the Wheat Seedling Stage

In plants, the phenylpropanoid biosynthesis pathway is suggested to be vital for adaptation to environmental stresses [35]. The accumulation of phenolic compounds through the activation of this pathway plays an important role in neutralizing harmful ROS and protecting the plant from oxidative damage caused by ROS [19]. Under waterlogging stress, the phenylpropanoid biosynthesis pathway was frequently enriched in different species, such as *Triarrhena sacchariflora* [7], alfalfa [10], wheat [9], cucumber [11], and *Phalaris arundinacea* [12]. Consistent with these studies, the phenylpropanoid biosynthesis pathway was significantly enriched under the three flooding conditions in this study (Figure 5), and 21 genes were co-upregulated by WL, HS, and FS, suggesting that the activation of the phenylpropanoid pathway may be an important mechanism underlying plant responses to flooding stress.

Three preliminary steps of the phenylpropanoid biosynthesis pathway, catalyzed by phenylalanine ammonia-lyase, 4-coumarate CoA ligase, and trans-cinnamate 4-monooxygenase, are essential for subsequent branching and regulating the production of antioxidant phenolic compounds such as flavonoids, lignins, and tannins. A previous study demonstrated that waterlogging induced the high expression of isochorismate synthase- and phenylalanine ammonia lyase-related genes in wheat and enhanced the SA content in the roots, which promoted the formation of axile roots and surface adventitious roots [36]. Consistent with this, a total of 7, 13, and 23 phenylalanine ammonia-lyase genes were upregulated by WL, HS, and FS, and 9 of these were uniquely regulated by FS (Figure 5B). Additionally, two and three 4-coumarate CoA ligase genes were uniquely upregulated and downregulated under the WL and FS treatments, respectively. Three genes encoding trans-cinnamate 4-monooxygenase were only downregulated under the FS condition (Figure 5B). Lignin is the main component of the plant cell wall and is one of the most important products in the phenylpropanoid biosynthesis pathway, which plays an important role in the response of wheat to waterlogging stress [37]. Caffeic acid O-methyltransferase and caffeoyl-CoA O-methyltransferase are two important enzymes that participate in lignin biosynthesis, controlling the syringyl and guaiacyl units of the lignin polymer, respectively [38]. The S/G ratio is demonstrated to be a major determinant of lignin quality [39]. The present investigation showed that three caffeic acid 3-O-methyltransferases were specifically upregulated under WL stress but downregulated under FS stress (Figure 5B). Two caffeoyl-CoA O-methyltransferase genes were uniquely upregulated under FS stress (Figure 5B). Shikimate O-hydroxycinnamoyltransferase is involved in the production of methoxylated monolignols, which are precursors to syringyl- and guaiacyl-unit lignins. In the present study, three DEGs encoding shikimate O-hydroxycinnamoyltransferases were only upregulated by FS (Figure 5B). Additionally, it was also observed that three 5-O-4-coumaroyl-D-quinate 3′-monooxygenases, which catalyze the conversion of p-coumaryl CoA to p-coumaryl shikimate, were uniquely downregulated under the FS condition (Figure 5B). These results suggest that the synthesis of different phenolic components may be an important mechanism of the plant response to different types of flooding. However, further research is needed to confirm this hypothesis.

### 3.4. Specific Responses of Plant Hormone Signaling Genes to Different Depths of Flooding Stress

Plant hormones, including IAA, CTK, GA, ABA, ETH, BRs, JA, and SA, play an important role in the stress resistance of many plants [39]. Many genes involved in plant hormone signaling were found to be affected in flooded plants [11,12,40]. ABA is widely recognized as an important hormone and chemical signal in plants responding to waterlogging stress [41]. Under waterlogging stress, stomatal closure, presumably regulated by abscisic acid (ABA), is triggered to prevent dehydration [42]. The *Arabidopsis* abi2-1 mutant, which has a higher stomatal transpiration due to impaired ABA signaling, showed enhanced plant survival after submergence [43]. Waterlogging stress can promote the accumulation of ABA, which leads to a reduction in stomatal conductance in leaves [7]. In the ABA signaling pathway, PYR/PYL, the most upstream regulator in this pathway, interacts with PP2C to reduce the inhibition of SnRK2, thereby regulating downstream factors such as ABF [44]. When plants are exposed to waterlogging, the DEGs regulating PYL are significantly activated and upregulated [39]. The level of endogenous IAA was increased in *Prunus persica* leaves under waterlogging stress [45]. AUX/IAA and SAUR are the two major classes of primary auxin response genes. The genes in the IAA signaling pathway are negatively regulated by waterlogging [8]. In plants, SA is a common phenolic compound. Under waterlogging conditions, the content of SA was significantly increased in waterlogging-tolerant soybean cultivars, further stimulating adventitious roots, enhancing gas exchange, and ultimately conferring tolerance to waterlogging stress [46]. Exogenous SA promotes the formation of axile roots and surface adventitious roots in wheat under waterlogged conditions [34]. Chen et al. (2022) found that a decreased SA content caused by low-expressed PAL might impact the resistance of *Styrax tonkinensis* seedlings to waterlogging stress [47]. Zhang et al. (2017) further suggested that JA, SA, and BR are involved in the waterlogging response, given that many differentially expressed genes associated with JA, SA, and BR were upregulated in stressed cotton plants [48]. Consistent with these studies, the plant hormone transduction pathway was significantly enriched in wheat seedlings under the three flooding conditions (Figure 6B and Table S8). Most of the DEGs associated with IAA, GA, ABA, BRs, JA, and SA were upregulated under one or more flooding conditions, highlighting the importance of plant hormones in the plant responses to flooding stress.

On the other hand, a large number of plant hormone signal-related genes were flooding-type-specific. For example, GA-, JA-, ETH-, and CTK-related DEGs were mainly induced by FS, and a few genes associated with ABA, SA, and IAA were coregulated under the three flooding conditions (Figure 6B and Table S20). We speculate that wheat responds to different types of flooding stress by activating different hormone signaling pathways.

### 3.5. Flooding-Type-Dependent Response Pathways and TFs

Previous research has suggested a possible connection between the number of responsive genes and their association with the complexity and intensity of the imposed stress treatment [49]. For example, experiments in barley exposed to combined water deficits and salt stress showed that the duration of individual or combined stresses increased the number of differentially expressed genes [49]. Consistent with these studies, the present study revealed that the negative effect of FS on the growth of wheat seedlings was greater than those of HS and WL (Figure 1A). At the same time, we observed clear differences in the numbers and types of DEGs that were upregulated with differences in water depth (Figure 3B,C). Furthermore, previous studies demonstrated that the response of plants to stress is related to the type of stress [49,50]. For instance, Luo et al. (2019) found that the CDPK, MAPK, CIPK, and PYL-PP2C-SnRK2 signaling pathways were involved in osmotic stress, while the SOS core pathway was activated by ionic stress [50]. Consistent with our study, a KEGG enrichment analysis indicated that several pathways were specially enriched in the WL, HS, and FS treatments, such as the ‘brassinosteroid biosynthesis’ pathway under the WL treatment, ‘taurine and hypotaurine metabolism’ under the HS treatment, and ‘carbon metabolism’ under the FS treatment (Figure 3E–G), suggesting that type-specific response pathways exist in plant responses to flooding stress.

Transcription factors (TFs) are crucial controllers of abiotic stress, including waterlogging, and participate in the regulation of downstream stress-responsive genes [7,9]. These proteins have been identified as among the most promising targets for the improvement of plant performance under waterlogging stress [7]. Here, a total of 1754 differentially expressed TFs from 35 different families were identified in wheat seedlings under WL, HS, and FS conditions (Figure 6C and Table S21). The greatest difference was the significant over-representation of the bHLH, NAC, MYB, WRKY, and AP2/ERF families in the upregulated DEGs and downregulated DEGs in response to flooding. Consistent with previous studies, these TF families are involved in abiotic stress and may positively improve plant tolerance [7,9]. In soybean, *Glyma.01G198000*, encoding a bHLH TF, was predicted to be a candidate gene involved in seed waterlogging tolerance [51]. The constitutive expression of the *TaERFVII.1* gene in wheat enhanced the tolerance to waterlogging in transgenic wheat without negative impacts on its development and yield [15]. However, in this study, the three copies of the *TaERFVII.1* gene (TraesCS5B02G315500, TraesCS5A02G314600, and TraesCS5D02G32080) were barely expressed under both control and flooded conditions (Table S2). This result suggests that wheat tolerance to flooding stress is regulated by other genes. Moreover, a large number of flooding-type specific TFs were also identified across the three flooding conditions; for example, two TF families, ARR-B and YAABY, were only identified under the FS treatment. Stress-type-specific-response TFs were also reported by Osthoff et al. (2019) and Luo et al. (2019), between the NaCl and mannitol treatments [49], and between the water deficit and salt treatments [50], respectively. These results may indicate that plants respond to stress via a complex regulatory mechanism by varying the combination and concentration of TFs according to the stress type.

### 3.6. Type-Dependent Alterations in DNA Methylation Levels under the WL, HS, and FS Treatments

Epigenetic regulators are remarkably diverse in plants, facilitating the phenotypic plasticity of plant development, survival and reproduction in unfavorable environments [52]. In plants, alterations in histone modification and DNA methylation are coordinated with changes in the expression of stress-responsive genes to adapt to environmental changes [53]. DNA methylation is a well-studied epigenetic mechanism underlying plant stress responses [20,21,54]. Furthermore, the response of DNA methylation to stress is related to the intensity and type of stress. For example, chromium stress increased the DNA methylation level in kenaf in a chromium-concentration-dependent manner [21]. Li et al. (2022) showed that heat stress increased the DNA methylation level in a heat-sensitive rice group but decreased it in a tolerant rice group [20]. The total DNA methylation in sesame increased under drought stress but decreased under waterlogging stress [17]. In this study, both WL and HS increased the total DNA methylation level in wheat seedlings, but this was decreased under FS (Table 1). Thus, we deduced that flooding-type-dependent alterations in DNA methylation levels may be a tolerance strategy in wheat. In future studies, it would be valuable to compare the differences in genome-wide methylation under different flooding conditions using high-throughput methylation sequencing.

## 4. Materials and Methods

### 4.1. Plant Material and Flooding Treatments

A commercial spring wheat (*Triticum aestivum* L.) variety Efumai 1 was used in this study. Seeds of Efumai 1 were surface-sterilized with 2% sodium hypochlorite for 15 min and rinsed with deionized water three times. The seeds were then planted in growth pots (6 plants in each pot), filled with the same amount of soil, and placed in a greenhouse with a 14 h/10 h and 22 °C/18 °C day/night and light/temperature cycle. Relative humidity was maintained at 70%. Six pots were established for each replicate. After 18 days of growth (at the stage with three fully expanded leaves), uniform seedlings were kept and divided into four groups, including the CK and three flooding treatments. The three types of flooding stress treatments were designed based on the depth of the water table, described as waterlogging (WL: keeping the water level at the surface soil level ±1 cm), half submergence (HS: keeping the water level at half of the plant height), and full submergence (FS: keeping the plant fully submerged). For the control, the seedlings were grown in ideal conditions. A completely randomized design with three biological replicates was applied in this experiment.

### 4.2. Physiological Measurements and Growth Parameters

After a 7-day treatment, the maximum photochemical efficiency of PSII (*F_v_/F_m_*) and relative chlorophyll content (SPAD) were measured in the first completely-extended top leaves using a MINI-PAM-II (Walz, Eiffeltrich, Germany) and a chlorophyll meter (Minolta SPAD-502, Tokyo, Japan), respectively, according to the manufacturer’s protocols. The upper, middle, and lower parts of the wheat leaves were measured twice, and the average value was taken as one repeat. Six plants were randomly selected for each biological repeat. Shoot (both stem and leaves) dry weight was measured for each treatment (10 plants of each treatment) after drying at 105 °C in an oven for 72 h.

On the other hand, the first top leaves of Efumai 1 were randomly selected and stored at −80 °C to further detect the physiological and molecular changes that occurred under the CK and three flooding conditions. H_2_O_2_ was extracted by homogenizing a 0.3 g sample with 2.7 mL of phosphate buffer (50 mM, pH 6.5) at 4 °C. The H_2_O_2_ concentration was determined via a colorimetric method using a commercial kit (Nanjing Jiancheng Bioengineering Institute, Nanjing, China), according to the manufacturer’s instructions. The absorbance value of each sample was calibrated to a standard concentration curve to calculate the H_2_O_2_ concentration. The results are expressed as mmol g^−1^ protein. The MDA content was extracted using a 5% trichloroacetic acid (TCA) buffer. The activities of enzymes (SOD, POD, and CAT) were extracted using a 50 mM potassium phosphate buffer (pH 7.8, containing 1% (*w*/*v*) polyvinylpolypyrrolidone (PVP), and 0.1 mM ethylenediaminetetraacetic acid (EDTA), and 0.3% (*w*/*v*) Triton X100), respectively. The absorbance of each sample was measured using a UV-1800 spectrophotometer (Shimadzu, Kyoto, Japan) and quantified according to the method described by Ren et al. (2020) [55]. MDA contents and enzyme activities are expressed as nmol g^−1^ FW and U g^−1^ FW, respectively.

### 4.3. Methylation-Sensitive Amplified Polymorphism (MSAP) Analysis

The total DNA of each sample was isolated using the CTAB procedure. The procedure of MSAP analysis, which uses two pairs of restriction endonucleases (*EcoR*Ⅰ + *Hpa*Ⅱ and *EcoR*Ⅰ + *Msp*Ⅰ) (Thermo Fisher Scientific, Waltham, MA, USA) for the restrictive digestion of the DNA of each sample, is described in previous research [20]. The adaptors and primers are listed in Table S22. The PCR products were separated using a Fragment Analyzer Automated CE System (AATI, Ames, IA, USA) and the DNF-900 dsDNA Reagent Kit 35~5500 bp (AATI, USA), according to Li et al. (2022) [20]. The MSAP data were exported using PROSize version 2.0 software (AATI, USA) and transformed into a binary character matrix, using “1” or “0” to indicate the presence or absence of bands. Only the consistent epilocus among the three biological repeats was used for future analysis. The three types of MSAP bands were defined as nonmethylation, hemi-methylation, and full methylation (Table 1) according to Tang et al. (2022) [21].

### 4.4. RNA Extraction, Library Preparation, and Sequencing

The total RNA of each sample was isolated using a TRNzol Universal RNA extraction Kit (Tiangen, Beijing, China) according to the manufacturer’s instructions. The quantity and purity of each RNA sample were determined using a Nanodrop 2000 (ThermoFisher Scientific, Waltham, Massachusetts, USA) and 2100 Bioanalyzer instrument (Agilent Technologies, Santa Clara, CA, USA), respectively. cDNA Library preparation for RNA-Seq was conducted using a NEBNext^®^ Ultra™ RNA Library Prep Kit for Illumina^®^ (NEB, Ipswich, MA, USA), following the manufacturer’s recommendations. The final library quality was assessed using the Agilent Bioanalyzer 2100 system (Agilent Technologies, USA), and the library was sequenced on an Illumina NovaSeq platform (150 bp pair end). Three biological replicates were used for RNA-Seq experiments.

### 4.5. RNA-Seq Read Mapping, Sequence Assembly, and Differential Expression

After filtering the sequencing adapters and low-quality reads, valid data were aligned to the wheat reference genome (https://urgi.versailles.inra.fr/download/iwgsc/IWGSC_RefSeq_Assemblies/v1.0/, accessed on 1 May 2023) using HISAT2, and the alignments were sorted using SAMTools v1.8 with default parameters. The uniquely mapped reads were aligned with high-confidence genome annotation files (IWGSC RefSeq v1.1, https://wheat-urgi.versailles.inra.fr/Seq-Repository/Annotations, accessed on 5 May 2023). The reads mapped to each gene were counted using featureCounts. The expression level of each gene was estimated from the fragments per kilobase of transcript per million fragments mapped (FPKM) value. Differential expression analysis between the CK and the treatments was performed using DESeq2 software (version 1.26.0). An adjusted *p* value < 0.05 and |log2-fold fold change| ≥1 were used as criteria for identifying differentially expressed genes (DEGs).

### 4.6. Functional Analysis of DEGs

Gene Ontology (GO) and Kyoto Encyclopedia of Genes and Genomes (KEGG) enrichment analyses of the DEGs were performed using the free online platform, OmicShare tools (http://www.omicshare.com/tools, accessed on 10 May 2023), where the threshold was a corrected *p* value < 0.05. Transcription factors (TFs) were predicted and classified into different families using PlantTFDB (http://planttfdb.gao-lab.org/, accessed on 11 May 2023). Heatmaps of gene expression were generated using TBtools (version v2.008) [56].

### 4.7. Quantitative Real-Time PCR (qRT–PCR) Validation

To validate the repeatability and reproducibility of the gene expression data obtained via RNA-Seq in the four conditions, a total of 20 genes were randomly selected, and gene-specific primers were designed with the online tool Primer 3 (http://primer3.ut.ee, accessed on 25 May 2023). First-strand cDNA was synthesized using the UEIris RT mix with a Dnase (All-in-One) kit (US Everbright, Jiangsu, China) according to the manufacturer’s protocol. qRT–PCR was performed with the QuantStudio™ 6 Flex Real-Time PCR System (Applied Biosystems, Foster, CA, USA) using Biolife Green I (US Everbright, Jiangsu, China). Actin was used as an internal control to normalize the gene expression level. The relative expression levels of selected genes were determined using the 2^−ΔΔCT^ method. The primers for qRT–PCR are presented in Table S23. All reactions were performed in triplicate.

### 4.8. Statistical Analysis

All statistical analyses were performed using SPSS 18.0. The data were analyzed using a one-way analysis of variance (ANOVA, Duncan’s test). Differences with *p* < 0.05 were considered significant.

## 5. Conclusions

Our research provides the first characterization of the physiological, epigenetic, and transcriptomic responses of wheat seedlings to different levels of flooding stress. The results suggest that there was no difference in the effects of WL and HS on biomass and physiology without the submergence of functional leaves. However, significant differences in epigenetic and transcriptional responses were identified among the three flooding conditions. FS increased the levels of H_2_O_2_ and MDA in the leaves and led to greater reductions in the chlorophyll content (SPAD), photochemical efficiency (*F_v_*/*F_m_*), and biomass production than HS and WL. Type-dependent alterations in DNA methylation were observed between the three flooding conditions. Similarly, the common and specific response pathways of wheat to the flooding stresses of different water depths were also observed. We found that the DEGs involved in ‘photosynthesis’, ‘phenylpropanoid biosynthesis’, and ‘plant hormone signal transduction’ were closely related to the flooding response, and these genes were involved in specific responses to different flooding stress types. Thus, our results provide a comprehensive view of the complex molecular events involved in the responses of wheat leaves to flooding stress, which will promote research on the development of flood-resistant crops and provide new information for wheat breeders. In the future, a systemic investigation of epigenetic regulation and flooding tolerance would be valuable. Moreover, flood depth is also a factor worth considering in future flood assessments.

## Figures and Tables

**Table 1:** MSAP-based cytosine methylation levels in wheat leaves under CK, WL, HS, and FS treatments.

MSAP Band Types	Patterns ^a^		CK	WL	HS	FS
	*Hpa*Ⅱ	*Msp*Ⅰ				
Ⅰ	1	1	53	49	30	60
Ⅱ	1	0	136	123	41	134
Ⅲ	0	1	181	151	158	156
IV	0	0	83	130	224	103
Total amplified bands			453	453	453	453
Hemi-methylated ratio (%) ^b^			30.02%	27.15%	9.05%	29.58%
Full methylated ratio (%) ^c^			58.28%	62.03%	84.33%	57.17%
Total methylated ratio (%) ^d^			88.30%	89.18%	93.38%	86.75%

^a^ The numbers ‘1’ or ‘0’ represent the presence or absence of bands, respectively. Type I (*Hpa*Ⅱ/*Msp*Ⅰ, 1/1) indicates unmethylation, type II (*Hpa*Ⅱ/*Msp*Ⅰ, 1/0) indicates hemi-methylation, and type Ⅲ (*Hpa*Ⅱ/*Msp*Ⅰ, 0/1) and type Ⅳ indicate full methylation. ^b^ Hemi-methylated ratio (%) = [(Ⅱ/(Ⅰ + Ⅱ + Ⅲ+ Ⅳ)] × 100. ^c^ Fully methylated ratio (%) = [(Ⅲ + Ⅳ)/(Ⅰ + Ⅱ + Ⅲ + Ⅳ)] × 100. ^d^ Total methylated ratio (%) = [(Ⅱ + Ⅲ + Ⅳ)/(Ⅰ + Ⅱ + Ⅲ + Ⅳ)] × 100.

**Table 2:** Alternations in DNA methylation patterns induced by the WL, HS, and FS treatments.

Description of Groups	CK		Flooding-Treated		WL	HS	FS
	*Hpa*Ⅱ	*Msp*Ⅰ	*Hpa*Ⅱ	*Msp*Ⅰ			
No change	1	1	1	1	30	16	29
	0	0	0	0	100	72	102
	1	0	1	0	29	22	10
	0	1	0	1	90	23	79
				Total	249 (56.21%)	133 (30.50%)	220 (48.89%)
Hypomethylation	1	0	1	1	12	7	16
	0	1	1	1	3	3	9
	0	0	1	1	4	4	6
	0	1	1	0	5	3	18
	0	0	1	0	16	11	24
	0	0	0	1	34	46	43
				Total	74 (16.70%)	74 (16.97%)	116 (25.78%)
Hypermethylation	0	1	0	0	73	103	52
	1	0	0	0	24	89	38
	1	1	0	0	4	10	3
	1	1	0	1	7	23	8
	1	1	1	0	12	4	13
				Total	120 (27.09%)	229 (52.52%)	114 (25.33%)

## Data Availability

The original contributions presented in the study are publicly available. All raw sequencing data have been submitted to the National Center for Biotechnology Information (BioProject: PRJNA826919).

## Supplementary Materials

The following supporting information can be downloaded at https://www.mdpi.com/article/10.3390/ijms242316785/s1.

## References

[1] Manik S.M.N., Quamruzzaman M., Livermore M., Zhao C.C., Johnson P., Hunt I., Shabala S., Zhou M.X. (2022). Impacts of barley root cortical aerenchyma on growth, physiology, yield components, and grain quality under field waterlogging conditions. Field Crops Res..

[2] Sasidharan R., Bailey-Serres J., Ashikari M., Atwell B.J., Colmer T.D., Fagerstedt K., Fukao T., Geigenberger P., Hebelstrup K.H., Hill R.D. (2017). Community recommendations on terminology and procedures used in flooding and low oxygen stress research. New Phytol..

[3] Manik S.M., Pengilley G., Dean G., Field B., Shabala S., Zhou M.X. (2019). Soil and crop management practices to minimize the impact of waterlogging on crop productivity. Front. Plant Sci..

[4] Xiao Y.S., Wu X.L., Sun M.X., Peng F.T. (2020). Hydrogen sulfide alleviates waterlogging-induced damage in peach seedlings via enhancing antioxidative system and inhibiting ethylene synthesis. Front. Plant Sci..

[5] Liu K., Harrison M.T., Shabala S., Meinke H., Ahmed I., Zhang Y.B., Tian X.H., Zhou M.X. (2020). The state of the art in modeling waterlogging impacts on plants: What do we know and what do we need to know. Earth’s Future.

[6] Liu K., Harrison M.T., Archontoulis S.V., Huth N., Yang R., Liu D.L., Yan H.L., Meinke H., Huber I., Feng P.Y. (2021). Climate change shifts forward flowering and reduces crop waterlogging stress. Environ. Res. Lett..

[7] Wang J., Sun H., Sheng J.J., Jin S.R., Zhou F.S., Hu Z.L., Diao Y. (2019). Transcriptome, physiological and biochemical analysis of *Triarrhena sacchariflora* in response to flooding stress. BMC Genet..

[8] Li Y., Shi L.C., Yang J., Qian Z.H., He Y.X., Li M.W. (2021). Physiological and transcriptional changes provide insights into the effect of root waterlogging on the aboveground part of *Pterocarya stenoptera*. Genomics.

[9] Shen C.W., Yuan J.P., Qiao H., Wang Z.J., Liu Y.H., Ren X.J., Wang F., Liu X., Zhang Y., Chen X.L. (2020). Transcriptomic and anatomic profiling reveal the germination process of different wheat varieties in response to waterlogging stress. BMC Genet..

[10] Zeng N.B., Yang Z.J., Zhang Z.F., Hu L.X., Chen L. (2019). Comparative transcriptome combined with proteome analyses revealed key factors involved in Alfalfa (*Medicago sativa*) response to waterlogging stress. Int. J. Mol. Sci..

[11] Keska K., Szczesniak M.W., Makalowska I., Czernicka M. (2021). Long-Term waterlogging as factor contributing to hypoxia stress tolerance enhancement in cucumber: Comparative transcriptome analysis of waterlogging sensitive and tolerant accessions. Genes.

[12] Wang X., He Y., Zhang C.B., Tian Y.A., Lei X., Li D.X., Bai S.Q., Deng X.G., Lin H.H. (2021). Physiological and transcriptional responses of *Phalaris arundinacea* under waterlogging conditions. J. Plant Physiol..

[13] Salah A., Zhan M., Cao C.G., Han Y.L., Ling L., Liu Z.H., Li P., Ye M., Jiang Y. (2019). Gamma-aminobutyric acid promotes chloroplast ultrastructure, antioxidant capacity, and growth of waterlogged maize seedlings. Sci. Rep..

[14] Yu F., Liang K., Fang T., Zhao H.L., Han X.S., Cai M.J., Qiu F.Z. (2019). A group VII ethylene response factor gene, *ZmEREB180*, coordinates waterlogging tolerance in maize seedlings. Plant Biotechnol. J..

[15] Wei X.N., Xu H.J., Rong W., Ye X.G., Zhang Z.Y. (2019). Constitutive expression of a stabilized transcription factor group VII ethylene response factor enhances waterlogging tolerance in wheat without penalizing grain yield. Plant Cell Environ..

[16] Wang W.S., Qin Q., Sun F., Wang Y.X., Xu D.D., Li Z.K., Fu B.Y. (2016). Genome-wide differences in DNA methylation changes in two contrasting rice genotypes in response to drought conditions. Front. Plant Sci..

[17] Komivi D., Marie A.M., Zhou R., Zhou Q., Yang M., Ndiaga C., Diaga D., Wang L.H., Zhang X.R. (2018). The contrasting response to drought and waterlogging is underpinned by divergent DNA methylation programs associated with transcript accumulation in sesame. Plant Sci..

[18] Pan R., Xu Y.H., Xu L., Zhou M.X., Jiang W., Wang Q., Zhang W.Y. (2020). Methylation changes in response to hypoxic stress in wheat regulated by methyltransferases. Russ. J. Plant Physiol..

[19] Kumar S., Bhushan B., Wakchaure G., Meena K.K., Kumar M., Meena N.L., Rane J. (2020). Plant phenolics under water-deficit conditions: Biosynthesis, accumulation, and physiological roles in water stress alleviation. Plant Phenolics in Sustainable Agriculture.

[20] Li B., Cai H.Y., Liu K., An B.Z., Wang R., Yang F., Zeng C.L., Jiao C.H., Xu Y.H. (2022). DNA methylation alterations and their association with high temperature tolerance in rice anthesis. J. Plant Growth Regul..

[21] Tang M.Q., Yue J., Huang Z., Hu Y.L., Li Z., Luo D.J., Cao S., Zhang H., Pan J., Wu X. (2022). Physiological and DNA methylation analysis provides epigenetic insights into chromium tolerance in kenaf. Environ. Exp. Bot..

[22] Rao L., Li S., Cui X. (2021). Leaf morphology and chlorophyll fluorescence characteristics of mulberry seedlings under waterlogging stress. Sci. Rep..

[23] Manzur M.E., Grimoldi A.A., Striker G.G. (2020). The forage grass *Paspalum dilatatum* tolerates partial but not complete submergence caused by either deep water or repeated defoliation. Crop Pasture Sci..

[24] Striker G.G., Kotula L., Colmer T.D. (2019). Tolerance to partial and complete submergence in the forage legume *Melilotus siculus*: An evaluation of 15 accessions for petiole hyponastic response and gas-filled spaces, leaf hydrophobicity and gas films, and root phellem. Ann. Bot..

[25] Appels R., Eversole K., Stein N., Feuillet C., Keller B., Rogers J., Pozniak C.J., Choulet F., Distelfeld A. (2018). Shifting the limits in wheat research and breeding using a fully annotated reference genome. Science.

[26] Herzog M., Fukao T., Winkel A., Konnerup D., Lamichhane S., Alpuerto J.B., Hasler S.H., Pedersen O. (2018). Physiology, gene expression, and metabolome of two wheat cultivars with contrasting submergence tolerance. Plant Cell Environ..

[27] Herzog M., Striker G.G., Colmer T.D., Pedersen O. (2016). Mechanisms of waterlogging tolerance in wheat: A review of root and shoot physiology. Plant Cell Environ..

[28] Wang W., Du J., Chen L., Zeng Y., Tan X., Shi Q., Pan X., Wu Z., Zeng Y. (2021). Transcriptomic, proteomic, and physiological comparative analyses of flooding mitigation of the damage induced by low-temperature stress in direct seeded early indica rice at the seedling stage. BMC Genom..

[29] Wang J., Zhang Y.X., Yan X.R., Guo J.P. (2020). Physiological and transcriptomic analyses of yellow horn (*Xanthoceras sorbifolia*) provide important insights into salt and saline-alkali stress tolerance. PLoS ONE.

[30] Cheng X.X., Yu M., Zhang N., Zhou Z.Q., Xu Q.T., Mei F.Z., Qu L.H. (2016). Reactive oxygen species regulate programmed cell death progress of endosperm in winter wheat (*Triticum aestivum* L.) under waterlogging. Protoplasma.

[31] Gill M.B., Zeng F., Shabala L., Zhang G.P., Yu M., Demidchik V., Shabala s., Zhou M.X. (2019). Identification of QTL related to ROS formation under hypoxia and their association with waterlogging and salt tolerance in barley. Int. J. Mol. Sci..

[32] Wang S.Y., Zhou H., Feng N.J., Xiang H.T., Liu Y., Wang F., Li W., Feng S.J., Liu M.L., Zheng D.F. (2022). Physiological response of soybean leaves to uniconazole under waterlogging stress at R1 stage. J. Plant Physiol..

[33] Fakih Z., Plourde M.B., Germain H. (2023). Differential participation of plant ribosomal proteins from the small ribosomal subunit in protein translation under stress. Biomolecules.

[34] Liu Y., Beyer A., Aebersold R. (2016). On the dependency of cellular protein levels on mRNA abundance. Cell.

[35] Cao B.l., Li N., Xu K. (2020). Crosstalk of phenylpropanoid biosynthesis with hormone signaling in Chinese cabbage is key to counteracting salt stress. Environ. Exp. Bot..

[36] Koramutla M.K., Tuan P.A., Ayele B.T. (2022). Salicylic acid enhances adventitious root and aerenchyma formation in wheat under waterlogged conditions. Int. J. Mol. Sci..

[37] Nguyen T.N., Son S., Jordan M.C., Levin D.B., Ayele B.T. (2016). Lignin biosynthesis in wheat (*Triticum aestivum* L.): Its response to waterlogging and association with hormonal levels. BMC Plant Biol..

[38] Yang G., Pan W.Q., Zhang R.Y., Pan Y., Guo Q.F., Song W.N., Zheng W.J., Nie X.J. (2021). Genome-wide identification and characterization of caffeoyl-coenzyme A O-methyltransferase genes related to the *Fusarium* head blight response in wheat. BMC Genom..

[39] Hodgson K.K., Perlo V., Furtado A., Choudhary H., Gladden J.H., Simmons B.A., Botha F., Henry R.J. (2021). Association of gene expression with syringyl to guaiacyl ratio in sugarcane lignin. Plant Mol. Biol..

[40] Wang D., Chen Q., Chen W., Guo Q., Xia Y., Wang S., Jing D., Liang G. (2021). Physiological and transcription analyses reveal the regulatory mechanism of melatonin in inducing drought resistance in loquat (*Eriobotrya japonica Lindl*.) seedlings. Environ. Exp. Bot..

[41] Zhang Y., Liu G., Dong H., Li C. (2021). Waterlogging stress in cotton: Damage, adaptability, alleviation strategies, and mechanisms. Crop J..

[42] González-Guzmán M., Gómez-Cadenas A., Arbona V. (2021). Abscisic acid as an emerging modulator of the responses of plants to low oxygen conditions. Front. Plant Sci..

[43] Bui L.T., Shukla V., Giorgi F.M., Trivellini A., Perata P., Licausi F., Giuntoli B. (2020). Differential submergence tolerance between juvenile and adult *Arabidopsis* plants involves the *ANAC017* transcription factor. Plant J..

[44] Yang Y.Y., Gao S.W., Su Y.C., Lin Z.L., Guo J.L., Li M.J., Wang Z.T., Que Y.X., Xu L.P. (2019). Transcripts and low nitrogen tolerance: Regulatory and metabolic pathways in sugarcane under low nitrogen stress. Environ. Exp. Bot..

[45] Ateeq M., Khan A.H., Zhang D., Alam S.M., Shen W., Wei M., Meng J., Shen X., Pan J., Zhu K. (2023). Comprehensive physio-biochemical and transcriptomic characterization to decipher the network of key genes under waterlogging stress and its recuperation in *Prunus persica*. Tree Physiol..

[46] Kim Y.H., Hwang S.J., Waqas M., Khan A.L., Lee J.H., Lee J.D., Nguyen H.T., Lee I.J. (2015). Comparative analysis of endogenous hormones level in two soybean (*Glycine max* L.) lines differing in waterlogging tolerance. Front. Plant Sci..

[47] Chen H., Wu Q., Ni M., Chen C., Han C., Yu F.Y. (2022). Transcriptome analysis of endogenous hormone response mechanism in roots of *Styrax tonkinensis* under waterlogging. Front. Plant Sci..

[48] Zhang Y.J., Kong X.Q., Dai J.L., Luo Z., Li Z.H., Lu H.Q., Xu S.Z., Tang W., Zhang D.M., Li W.J. (2017). Global gene expression in cotton (*Gossypium hirsutum* L.) leaves to waterlogging stress. PLoS ONE.

[49] Osthoff A., Dalle R.P.D., Baldauf J.A., Piepho H.P., Hochholdinger F. (2019). Transcriptomic reprogramming of barley seminal roots by combined water deficit and salt stress. BMC Genom..

[50] Luo D., Zhou Q., Wu Y.G., Chai X.T., Liu W.X., Wang Y.R., Yang Q.C., Wang Z.Y., Liu Z.P. (2019). Full-length transcript sequencing and comparative transcriptomic analysis to evaluate the contribution of osmotic and ionic stress components towards salinity tolerance in the roots of cultivated alfalfa (*Medicago sativa* L.). BMC Plant Biol..

[51] Sharmin R.A., Karikari B., Chang F.G., Al Amin G.M., Bhuiyan M.R., Hina A., Lv W., Chunting Z., Begum N., Zhao T.J. (2021). Genome-wide association study uncovers major genetic loci associated with seed flooding tolerance in soybean. BMC Plant Biol..

[52] Kim J.M., Sasaki T., Ueda M., Sako K., Seki M. (2015). Chromatin changes in response to drought, salinity, heat, and cold stresses in plants. Front. Plant. Sci..

[53] Kim J.H. (2021). Multifaceted chromatin structure and transcription changes in plant stress response. Int. J. Mol. Sci..

[54] Samantara K., Shiv A., De-sousa L.L., Sandhu K.S., Priyadarshini P., Mohapatra S.R. (2021). A comprehensive review on epigenetic mechanisms and application of epigenetic modifications for crop improvement. Environ. Exp. Bot..

[55] Ren Y.F., Wang W., He J.Y., Zhang L.Y., Wei Y.J., Yang M. (2020). Nitric oxide alleviates salt stress in seed germination and early seedling growth of pakchoi (*Brassica chinensis* L.) by enhancing physiological and biochemical parameters. Ecotoxicol. Environ. Saf..

[56] Chen C.J., Chen H., Zhang Y., Thomas H.R., Frank M.H., He Y.H., Xia R. (2020). TBtools: An integrative toolkit developed for interactive analyses of big biological data. Mol. Plant.

