# Supplementary material for: Physiological, Epigenetic, and Transcriptome Analyses Provide Insights into the Responses of Wheat Seedling Leaves to Different Water Depths under Flooding Conditions
Source: Int J Mol Sci. 2023 Nov 26;24(23):16785. doi: 10.3390/ijms242316785 (PMC10706670; doi:10.3390/ijms242316785)
Supplement: Supplementary file 1 [file ijms-24-16785-s001.zip › Supplemental figures.pdf]

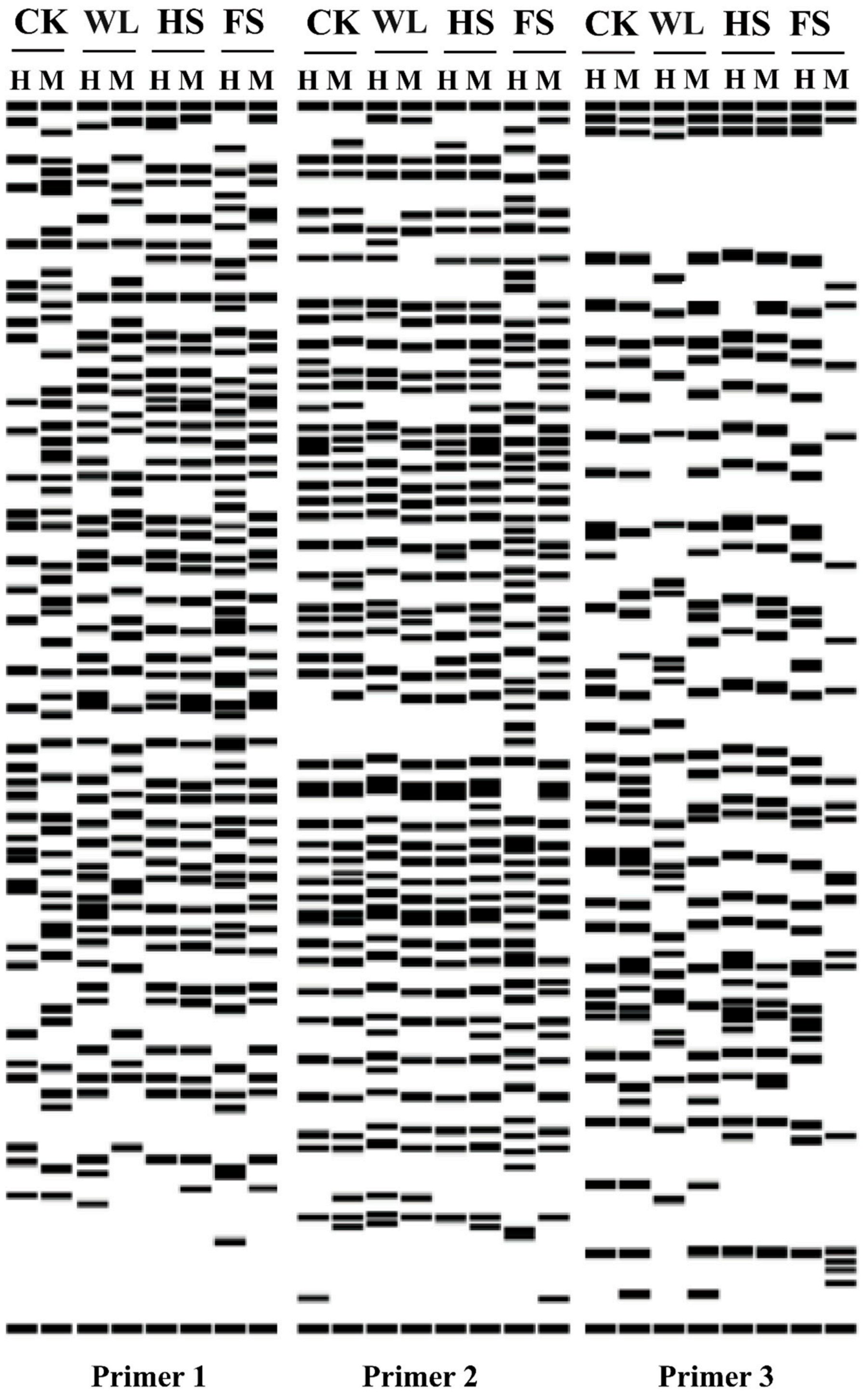

Figure S1. Partial MSAP bands monitored by capillary gel electrophoresis. H and M represent genomic DNA sample digested by *Eco*RI / *Hpa*II and *Eco*RI / *Msp*I combinations, respectively. MSAP markers generated with the selected primer combinations of *Hpa*II/*Msp*I + CTG / *Eco*RI + GTT (primer combination 1), *Hpa*II/*Msp*I + CTG / *Eco*RI + AAC (primer combination 2) and *Hpa*II/*Msp*I + TCCA / *Eco*RI + TA (primer combination 3).

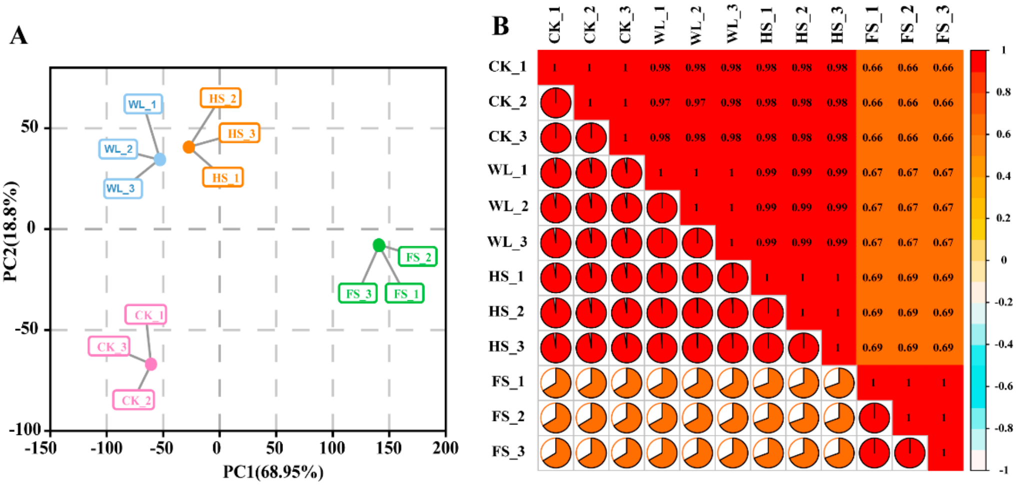

Figure S2. Principal component (A) and correlation analysis between biological replicates (B).

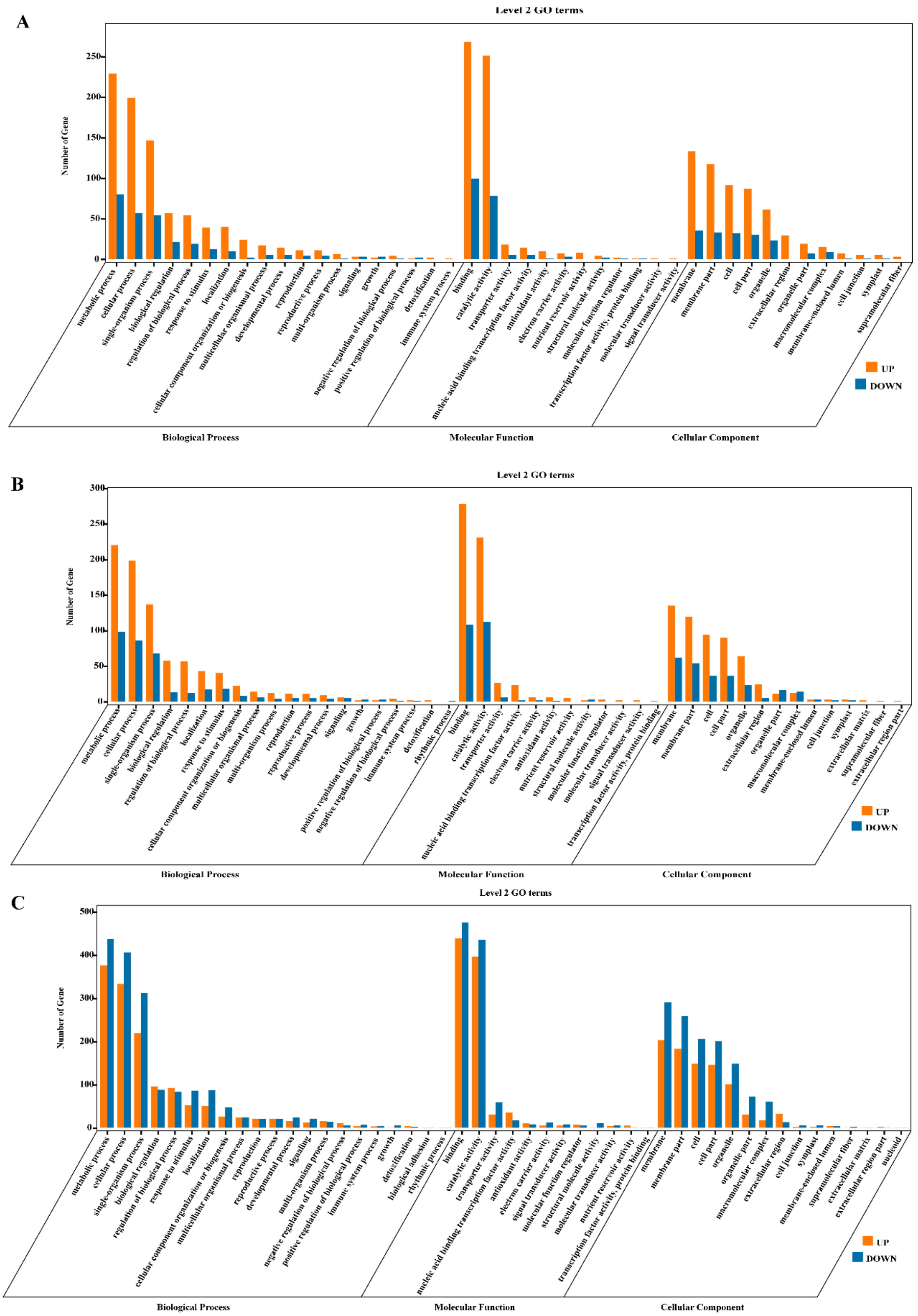

Figure S3 GO classification analysis of DEGs induced by WL (A), HS (B) and FS (C) treatment.
